# Characteristics of Mg-Based Sintered Alloy with Au Addition

**DOI:** 10.3390/ma16051915

**Published:** 2023-02-25

**Authors:** Sabina Lesz, Małgorzata Karolus, Adrian Gabryś, Bartłomiej Hrapkowicz, Witold Walke, Wojciech Pakieła, Klaudiusz Gołombek, Julia Popis, Peter Palček

**Affiliations:** 1 Department of Engineering Materials and Biomaterials, Silesian University of Technology, 18a Konarskiego Street, 44-100 Gliwice, Poland; 2 Institute of Materials Engineering, University of Silesia, 1a 75 Pulku Piechoty Street, 41-500 Chorzow, Poland; 3 Department of Biomaterials and Medical Device Engineering, Silesian University of Technology, Roosevelta 40 Street, 41-800 Zabrze, Poland; 4 Materials Research Laboratory, Silesian University of Technology, 18a Konarskiego Street, 44-100 Gliwice, Poland; 5 Department of Materials Engineering, Faculty of Mechanical Engineering, University of Žilina, Veľký Diel, SK-010 26 Žilina, Slovakia

**Keywords:** magnesium alloys, mechanical alloying, spark plasma sintering

## Abstract

The magnesium-based alloys produced by mechanical alloying (MA) are characterized by specific porosity, fine-grained structure, and isotropic properties. In addition, alloys containing magnesium, zinc, calcium, and the noble element gold are biocompatible, so they can be used for biomedical implants. The paper assesses selected mechanical properties and the structure of the Mg_63_Zn_30_Ca_4_Au_3_ as a potential biodegradable biomaterial. The alloy was produced by mechanical synthesis with a milling time of 13 h, and sintered via spark-plasma sintering (SPS) carried out at a temperature of 350 °C and a compaction pressure of 50 MPa, with a holding time of 4 min and a heating rate of 50 °C∙min^−1^ to 300 °C and 25 °C∙min^−1^ from 300 to 350 °C. The article presents the results of the X-ray diffraction (XRD) method, density, scanning electron microscopy (SEM), particle size distributions, and Vickers microhardness and electrochemical properties via electrochemical impedance spectroscopy (EIS) and potentiodynamic immersion testing. The obtained results reveal the compressive strength of 216 MPa and Young’s modulus of 2530 MPa. The structure comprises MgZn_2_ and Mg_3_Au phases formed during the mechanical synthesis, and Mg_7_Zn_3_ that has been formed during the sintering process. Although MgZn_2_ and Mg_7_Zn_3_ improve the corrosion resistance of the Mg-based alloys, it has been revealed that the double layer formed because of contact with the Ringer’s solution is not an effective barrier; hence, more data and optimization are necessary.

## 1. Introduction

Mechanical Alloying (MA) is a solid-state milling process which aims to obtain a fine, homogeneous powder. The input material comprises pure elements in selected proportions, or alloys, that are subjected to the high-energy milling in a special mill. It may be used for preparation of amorphous materials, as well as to change the phase composition, affecting the microstructure. The introduced materials are repeatedly crushed and agglomerated. Because of the cyclic deformations, i.e., welding, crushing, and re-welding, the grain size is reduced, and new grain boundaries are formed. The structure of the material is not stable. The alloy structure can be composed of either solid solutions or intermetallic or amorphous phases [1,2,3].

During the process, a mechanically induced reaction takes place between the powdery components of the alloy. This results in a change in the phase composition and microstructure. A special feature that distinguishes the MA process from the many ball milling processes is the occurrence of both crushing and melting processes [4].

Thanks to MA, it is possible to produce materials that are unobtainable, or very complicated to obtain, via traditional methods such as casting. Furthermore, it is also possible to achieve materials with a high purity and a strictly defined chemical composition. The MA process with the above-mentioned features enables the production of various components, including those for biomedical implants, as well as high-entropy alloys [4].

The biomaterials produced in this way have unique mechanical and surface properties like a human bone, so they can be considered as the future generation of biomaterials [5]. One of the most frequently used methods of improving the mechanical and biological properties of magnesium-based materials is the modification of their chemical composition. The selection of appropriate alloying additives and control of the content of impurities are key to improving the corrosion resistance of magnesium alloys [5].

Using the method of MA, it is possible to obtain a biocompatible Mg–Zn–Ca alloy with a nominal composition of 60 at. % Mg—35 at. % Zn—5 at. % Ca [6,7]. The addition of zinc increases the corrosion resistance of the alloy, and calcium is the main component of the mineralized tissue [7]. The addition of zinc in magnesium alloys causes significant changes in the microstructure of the alloy. It reduces the α-Mg grain size and thus significantly affects the corrosion resistance by controlling the alloy microstructure [8]. In the biological world, calcium is one of the important macronutrients. It is a component of bone-building hydroxyapatite. In the ionic form, it is necessary for the proper functioning of, among others, muscle tissue. It is involved in blood clotting, conduction of electrical impulses in the nervous system, and in immune reactions [9]. Depending on the structure, the alloy degrades with different rates of corrosion, hence featuring various biocompatibility. Materials produced by mechanical synthesis feature lower cytotoxicity than materials produced by casting [6,7].

The addition of noble metals to Mg–Zn–Ca alloys improves their properties. Gold has been used for centuries as a biomaterial, although today it has few medical applications. Its largest application area is in jewelry, in dentistry, and in brachytherapy, although new applications may yet appear in the near future by utilization of nanoparticles for drug release and sensor systems. Au is generally considered an inert metal and is more common in, e.g., biosensors because of its electrical conductivity and low solubility. It is also known for its immunity to corrosion [10]. The cytotoxic effect of Au is negligible as it is commonly used, e.g., in jewelry and even in food. However, salts of Au are known to be of immunological and cytotoxic relevance [11].

It can be used for many medical applications. Gold implants are used in reconstructive surgery, drug delivery microchips, treatment of rheumatoid arthritis, and endovascular stents [12]. In the case of stents, Au is used as a coating, which increases their biocompatibility and hemocompatibility [10]. Moreover, gold in alloys with other precious and non-precious metals is also used for dental items such as crowns and bridges [13].

The research carried out on the bioresorbable Ca-Mg-Zn-Yb-B-Au alloy shows that the addition of B and Au to the alloy increases the corrosion resistance. Their presence slows down pitting corrosion in Ringer’s solution by creating a barrier between Ca, B, and Cl ions. Used Ringer’s solution was prepared from Millipore tablets with the following composition: NaCl: 1.125 g/tablet; KCl: 0.0525 g/tablet; CaCl_2_, anhydrous: 0.03 g/tablet; NaHCO_3_: 0.025 g/tablet. The EIS parameters for the Ca_32_Mg_12_Zn_38_Yb_18-x_B_x_Au_x_ alloys (x = 1, 2) were significantly better than for the Ca_55_Mg_20_Zn_25_, Ca_65_Mg_10_Zn_25_, and Ca_32_Zn_38_Mg_12_Yb_18_ alloys. The controlled rate of H_2_ evolution was the result of the variability of the amount of B and Au in the chemical composition of the examined alloys. The compressive strength and microhardness of the alloys were also improved by adding B and Au [13].

The effect of Au and Cu addition on the Mg–Zn–Ca was investigated in [14]. The analysis of the corrosion results allowed to describe the influence of 0.5 and 1% at. Au and Cu to corrosion resistance in artificial physiological fluid. Mg_69_Zn_25_Ca_5_Au_0.5_Cu_0.5_ and Mg_69_Zn_25_Ca_5_Cu_1_ metallic glasses indicated lower corrosion resistance compared to the Mg_69_Zn_25_Ca_5_Au_1_ alloy. The increase in Cu content resulted in an increase in the volume of released hydrogen and high cathodic activity. The results of the corrosion tests [14] showed that the corrosion current density and the hydrogen volume decreased more effectively after the addition of Au than Cu. The Mg_69_Zn_25_Ca_5_Au_1_ alloy has better overall corrosion resistance compared to the Mg–Zn–Ca alloys [14].

The addition of noble metals other than gold also improves the properties of the magnesium alloys. Ramya and Ravi [15] investigated the cast Mg_66_Zn_30_Ca_4_ and Mg_64_Zn_29_Ca_5_Ag_2_ alloys based on a thermodynamic model using the PHHS parameter with the principal effects of electron transfer, atom size mismatch, and randomness. The in vitro corrosion behavior of Mg_66_Zn_30_Ca_4_ and Mg_64_Zn_29_Ca_5_Ag_2_ in a simulated body fluid (SBF) solution analyzed by electrochemical tests showed that, on a comparable scale, the corrosion resistance of Mg_64_Zn_29_Ca_5_Ag_2_ nanocrystalline samples was higher than their amorphous Mg_66_Zn_30_Ca_4_ counterparts. The addition of Ag to the Mg–Zn–Ca alloy leads to the formation of corrosive phases such as MgAg, Zn_8_Ag_5_, and MgZnAg_2_, along with Ag oxides and hydroxides. Such phases contribute to the improvement of the corrosion resistance due to the passive layer formation. The increase in hardness is attributed to the lack of free volume in the nanocrystalline Mg_64_Zn_29_Ca_5_Ag_2_ alloy compared to the amorphous Mg_66_Zn_30_Ca_4_. Obtaining the appropriate combination of mechanical properties and corrosion resistance of Mg_64_Zn_29_Ca_5_Ag_2_ can be further tested for antibacterial properties and cytocompability to make them ideal materials for bioimplants [15].

Yu et al. [16] produced Mg–3Zn–0.2Ca–xAg alloys (x = 0%, 0.1%, 0.3%, 0.5%, 0.7%, wt.%) to improve the complex properties of Mg–3Zn–0.2Ca (wt.%) alloys. The microstructure shows the alloy has a relatively fine grain size of 2.08 µm with an Ag addition of 0.3 wt.%. Mg–3Zn–0.2Ca–0.3Ag alloy also has better mechanical properties, the elongation, yield point, and tensile strength than Mg–3Zn–0.2Ca, which are 4.57%, 5.7%, and 2.4%, respectively. Electrochemical experiments have shown that Mg–3Zn–0.2Ca–0.3Ag has a lower corrosion current density (30.5 A/cm^2^). After immersion in simulated body fluids at 37 °C for 15 days, the hydrogen release rate and the degradation rate of Mg–3Zn–0.2Ca–0.3Ag are significantly lower than that of Mg–3Zn–0.2Ca alloy. Moreover, the cytotoxicity test with L 929 cells shows that the Mg–3Zn–0.2Ca–0.3Ag alloy is biocompatible [16].

González et al. [17] investigated the effect of partial substitution of Mg by Pd on the microstructure, mechanical properties, and corrosion resistance of Mg_72-x_Zn_23_Ca_5_Pd_x_ alloys (x = 0, 2 and 6 at. %) synthesized by casting. While the Mg_72_Zn_23_Ca_5_ alloy is mainly amorphous, the addition of Pd reduces the glass-forming ability, thus promoting the formation of crystalline phases. The hardness of the tested alloys increases with the addition of Pd, from 2.71 GPa for x = 0 to 3.9 GPa for x = 6, mainly due to the formation of high-strength phases. In turn, the wear resistance is maximized for an intermediate Pd content (i.e., Mg_70_Zn_23_Ca_5_Pd_2_). Corrosion tests in simulated body fluid (Hank’s solution) indicate that Pd shifts the corrosion potential towards more positive values, thus delaying the biodegradability of this alloy. Moreover, since cytotoxic studies on mouse preosteoblasts show no dead cells after culture for 27 h, these alloys are potential candidates for use as biomaterials [17].

Spark Plasma Sintering (SPS) is a method of fast sintering of powder materials. The powder is heated using periodically repeated DC pulses, lasting from a few to several hundred milliseconds of low voltage but high intensity (from several to tens of thousands of amperes). The SPS process is characterized by a high efficiency factor due to the direct supply of energy to the sintered powder without energy losses from heating the environment. The duration of the process is from several seconds to several minutes. Fast heating and cooling (up to 1000 °C/min) and short sintering time prevent the grains from excessive growth, allowing to preserve starting material microstructure, which is especially important in the case of sintering powders with nanometric or ultra-fine grain sizes [18].

Kumar [19] investigated biodegradable alloys based on Mg i.e., Mg–Zn–Mn–Si, Mg–Zn–Mn–HA, and Mg–Zn–Mn–Si–HA for bone stabilizing devices, produced with the SPS technique assisted by MA. The effects of hydroxyapatite, sintering temperature, and sintering pressures were assessed. Hydroxyapatite (HA) was added to the Mg matrix to modify the morphology, which resulted in the observation of coarse porous Mg with HA morphology. Several biocompatible intermetallic phases such as Ca–Mg, Mg–Zn, Mn–CaO, Mn–P, Ca–Mn–O, and ZnO_2_ have been produced in Mg with HA implants, which are beneficial for improving corrosion properties and bioactivity. During sintering, the formation of biomimetic oxide phases of the pore layer increased corrosion resistance and bioactivity. In addition, clinical trials are necessary to fulfill all claims regarding the statistical analysis of in vivo results. Porous and biodegradable structures have been successfully developed using the SPS technique for bone fixation devices. The developed alloy structures had properties close to the bone properties, with reasonable modulus of elasticity (29–45 GPa) and hardness (86–200 HV) [19].

The usage of noble metals, gold in this case, as an alloying addition to the modern biodegradable materials contrasts with the solutions used nowadays. The biodegradability of the proposed solution also greatly contributes as a factor to decrease patient discomfort. In addition, the current biomaterials are usually characterized by considerable energy consumptions and manufacturing limitations. Material proposed in this manuscript can tackle those issues with the use of MA and SPS, which facilitates the overall production as well as allows to prepare the desired set of parameters carefully.

This article presents a characteristic of the structure and properties of the Mg_63_Zn_30_Ca_4_Au_3_ powder produced by mechanical synthesis and the SPS method. Both for powders and sintered samples, the following were carried out: phase composition tests using X-ray analysis, morphology tests of the obtained powders and chemical composition using a high-resolution scanning electron microscope, microhardness measurements using the Vickers method. Additionally, for the powders, laser particle size analysis was performed as well as compressive strength tests and corrosion resistance tests for sintered samples.

## 2. Materials and Methods

The Mg_63_Zn_30_Ca_4_Au_3_ alloy was produced by MA using SPEX 8000D Mill (Metuchen, NJ, USA). First, 100 g of balls with a diameter of 10 mm made of 316L steel were used for milling. The ratio of the weight of balls to the weight of the powder was 10:1. The alloy was milled for 13 h. After each hour of milling the powder, there was a 30 min pause to prevent the powder particles from sticking to the container walls (cold welding) [5,20].

The milling time was selected based on previous studies [21,22], which show that the grinding time starting the amorphization process in the Mg_60_Zn_35_Ca_5_ alloy is 13 h.

The Mg–Zn–Ca–Au alloy was subjected to spark plasma sintering using a HP D 25/3 machine (FCT Systeme, Rauenstein, Germany). The sintering was carried out at a temperature of 350 °C and a compaction pressure of 50 MPa, with a holding time of 4 min and a heating rate of 50 °C∙min^−1^ to 300 °C and 25 °C∙min^−1^ from 300 to 350 °C. Samples were covered in graphite foil (Papyex N998 graphite foil, MERSEN, Gennevilliers, France) to increase the contacts conductivity and prevent sticking. After sintering the samples were sandblasted with silica sand (0.1–0.4 mm) to completely remove the graphite foil separating them from the SPS toolset.

The X-ray diffraction, Scanning Electron Microscopy, microhardness, and particle size distribution (granulometry) tests were carried out for the powders. For those sintered, compression tests were conducted as well.

First, the material was tested for phase composition using X-ray analysis on the PANalytical Empyrean Diffractometer (Almelo, The Netherlands) with Cu-Kα radiation (λ Kα1 = 1.5418 Å) and the PIXcell detector. Phase analysis was performed using the HighScore Plus 3.0 PANalytical software integrated with the ICDD PDF4 + 2016 crystallographic database. The structural characteristics of the sintered alloy and the size of crystallites determination were carried out using the Rietveld method implemented in the High Score Plus PANalytical software [23].

The morphology of the obtained powders and their chemical composition were examined using the HRSEM SUPRA 35 high-resolution scanning electron microscope (SEM) by the Zeiss company (Jena, Germany), equipped with an EDS detector.

Microhardness measurements were conducted using the Vickers method on the FM700 Vickers hardness tester (Future-Tech, Tokyo, Japan). The hardness measurement was carried out at 50 gf load (HV0.05) and dwell time 15 s [24]. The testing was performed in randomly selected areas of the prepared alloy to ensure statistically relevant mean hardness values.

Then, selected powder samples were subjected to laser particle size analysis on a Fritsch ANALYSETTE 22 MicroTec plus (Weimar, Germany) device. The particle size measurement test was performed in ethyl alcohol. The results of the grain composition analysis are presented as charts (histogram—percentage of grain content, the size of which falls in selected class compartments; cumulative curve—a continuous function illustrating the content in the studied material grains with smaller diameters, or larger than the selected diameter of D; grain distribution curve—diversified cumulative curve equivalent to the statistical density function). D_50_ (median) is used, among others, to characterize the grain size distribution.

The produced sintered samples were subjected to X-ray analysis and observed on a SEM. Their microhardness was also tested. Sintered samples were tested in a compression test on a Zwick Z020 testing machine (Zwick Roell Group, Ulm, Germany) according to EN ISO 3327 [25]. Samples were prepared with a base-to-height ratio of approximately 1:1.5 in cylindrical form. The result of the tests was plots of the relation between compressive strength and deformation of the sample. A compression test was used to determine compressive strength and the Young’s modulus. All the results were presented as the mean value. The samples were loaded to fracture at a rate of 2 mm∙min^−1^ at room temperature.

Corrosion resistance tests were carried out on the sinters. Pitting corrosion resistance was tested using the potentiodynamic method. To obtain information about the electrochemical properties of the surfaces of the analyzed samples, tests were also carried out with the use of electrochemical impedance spectroscopy (EIS). During the pitting resistance test, the anodic polarization curves were recorded using the PGP201 potentiostat from Radiometer (Copenhagen, Denmark) included in the measurement kit. A saturated calomel electrode of the KP-113 type was used as the reference electrode. The auxiliary electrode was a platinum electrode of the PtP-201 type. The research began with the determination of the E_OCP_ opening potential. Subsequently, the anodic polarization curves were recorded, taking measurements from the potential with the value E_start_ = E_OCP_ − 100 mV. The potential change occurred in the anode direction at a rate of 3 mV/s until the anode current density was 10 mA/cm^2^. Based on the recorded curves, characteristic values describing the resistance to pitting corrosion were determined, i.e., the corrosion potential E_corr_ [V], the polarization resistance R_p_ [Ω∙cm^2^], and the corrosion current density i_corr_ [A/cm^2^]. The Stern method was used to determine the value of the polarization resistance R_p_ (slope of the straight line). It was assumed that the β values for cathode and anode reactions are the same and amount to 0.12 V. The corrosion current density was determined from the simplified relationship i_corr_ = 0.026/R_p_.

EIS measurements were carried out using the Metrohm Auto Lab PGSTAT 302N (Herisau, Switzerland) measurement system equipped with the FRA2 (FRA—frequency response analyzer) module. The applied measuring system allowed to conduct tests in the frequency range from 10^4^ ÷ 10^−2^ Hz. The amplitude of the sinusoidal voltage of the excitation signal was 10 mV. In the research, impedance spectra of the system were determined, and the obtained measurement data were adjusted to the equivalent circuit. On this basis, numerical values of resistance R, capacitance C, and induction L of the analyzed systems were determined. The obtained EIS spectra were interpreted after fitting by the least squares method to the equivalent electrical system. All electrochemical tests were carried out in Ringer’s physiological solution at the temperature T = 37 ± 1 °C and pH = 6.8 ± 0.2.

## 3. Results and Discussion

### 3.1. X-ray Diffraction

**Figure 1:**
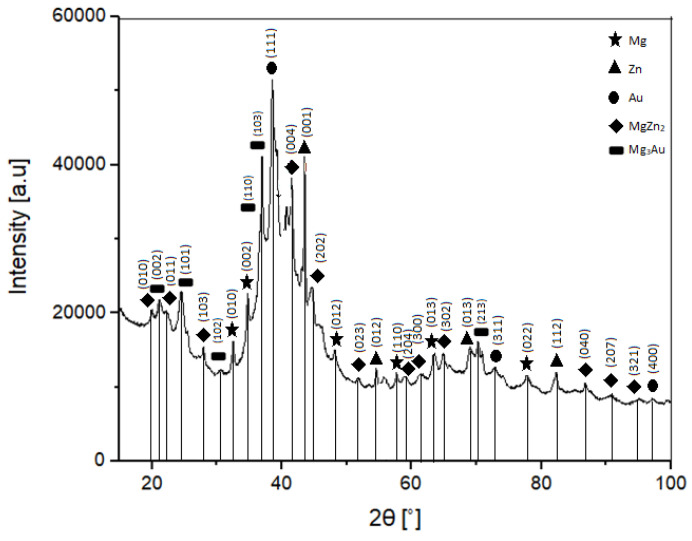
X-ray diffraction pattern of the Mg–Zn–Ca–Au alloy sample after 13 h milling.

Figure 1 shows the XRD pattern of the Mg–Zn–Ca–Au sample after 13 h of milling. The broadening of the XRD peak between 30 ÷ 50° 2θ suggests the presence of the amorphous phase and nano sized crystals. The identified phases consist of MgZn_2_, Mg_3_Au, Mg—as a base solid solution—and unreacted Zn residue. All identified phases are characterized by the P6_3/mmc_ space group. The diffraction pattern shows the progressing process of material amorphization, in which wide overlapping reflections together with the visible effect of diffusion scattering create a diffraction pattern characteristic of materials containing an amorphous component.

**Figure 2:**
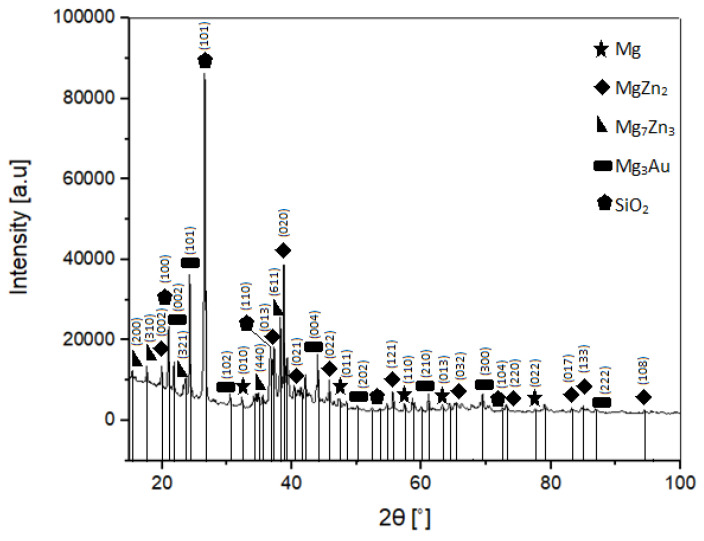
X-ray diffraction pattern of the Mg–Zn–Ca–Au sintered alloy.

Figure 2 shows the phase analysis of the sintered Mg–Zn–Ca–Au alloy. After sintering, the phase content in the samples changed partially. In addition to the phases present in the initial powder, i.e., a solid solution based on Mg, Zn, MgZn_2_, and Mg_3_Au, a new phase Mg_7_Zn_3_ (00-008-0269) appeared. A phase transition of the hexagonal MgZn_2_ phase (01-073-2566) to the monoclinic phase (04-008-7744) was also observed. The SiO_2_ phase visible in the diffractogram is an impurity coming from technological processes.

The size of the crystallites of the main phases identified in the material, i.e., MgZn_2_ and Mg(X) magnesium-based solid solution (where X = Zn, Ca, Au), is at the level of 350–360 Å, and the parameters of the elementary cells of these phases slightly increase (by 7% and 1%, respectively (Table 1).

The MgZn_2_ Laves phase is much stiffer and has greater shear resistance than pure magnesium, as is reported by Xie and Wu [26,27,28]. The Mg_7_Zn_3_ phase is even stronger than MgZn_2_. The Mg–Zn binary phase diagram indicates the Mg_7_Zn_3_ phase is a eutectic phase present over 325 °C at 30 at.% Zn content. However, the MgZn_2_ phase appears at high Zn content, meaning there is significant diffusion occurring during MA as well as sintering, as the phase is prevalent in both diagrams.

Their presence in the sintered material is very beneficial, as they improve the overall mechanical properties of the alloy. However, the properties of both the MgZn_2_ and Mg_7_Zn_3_ phases are still being researched.

The Mg_3_Au phase is a highly stable phase, which is confirmed by its presence in both powder and sintered diffraction patterns (Figure 1 and Figure 2). The temperature of the sintering process did not affect the Mg–Au phase, unlike the Mg–Zn phase, where the sintering temperature causes diffusion and formation of the Mg_7_Zn_3_ phase next to the already existing MgZn_2_ phase. In addition, it should be noted that the stability of the Mg–Au phases is also reported by Ferro [29]. This is due to the fact that Au alloys with elements of the second group are characterized by strongly negative values of the enthalpy of formation.

### 3.2. Scanning Electron Microscopy

**Figure 3:**
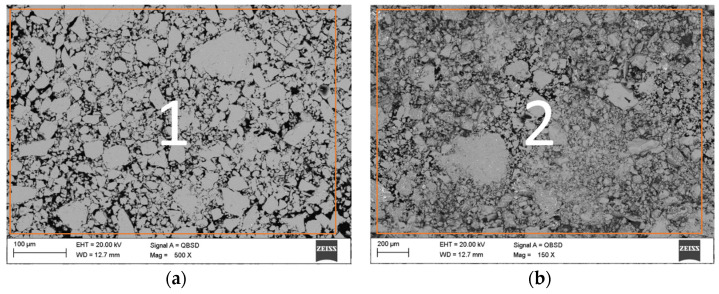
The SEM images of the Mg_63_Zn_30_Ca_4_Au_3_ powder after 13 h milling. Both images represent powder samples with different magnifications: (**a**) 500× and (**b**) 150×, the number in the images refer to the analysis results presented in Table 2.

Figure 3 indicates the SEM image together with the results of EDS analysis achieved for the powder after 13 h of milling time Figure 3 includes the SEM micrograph of the Mg63Zn30Ca4Au3 powder after 13 h of milling. Single grains have a globular and lamellar shape. Based on the chemical composition analysis of these grains, it can be concluded that they consist of magnesium, zinc, calcium, and gold (Table 2 and Table 3). The size of powder particles ranges between 9 to 60 μm on average.

**Figure 4:**
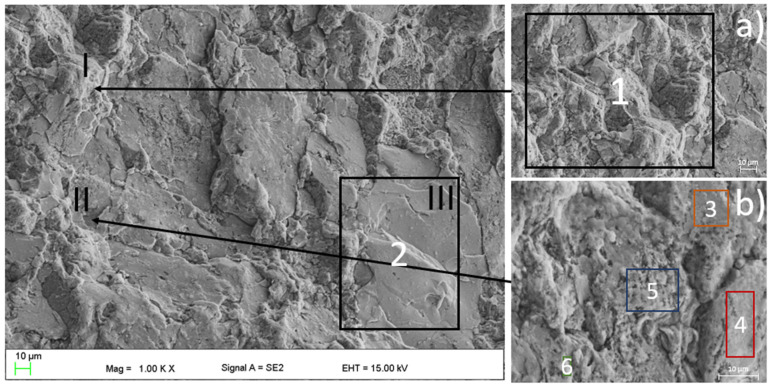
The SEM image with featured regions of the EDS analysis (marked with the Arabic numerals, the EDS values shown in the Table 2) obtained for the sintered Mg_63_Zn_30_Ca_4_Au_3_ alloy. Area marked with “I” and “II” refers to the (**a**) and (**b**) images, respectively, where the intercrystalline fractures can be seen. The region marked as “III” is presented in Figure 5.

**Figure 5:**
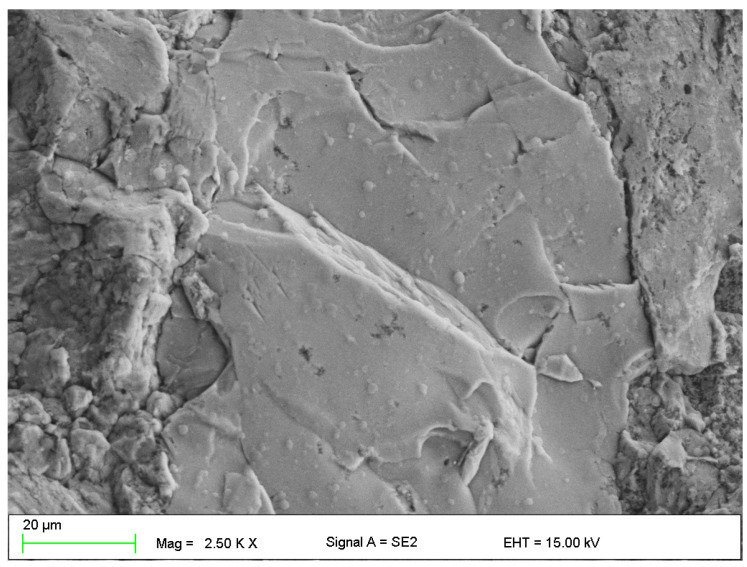
The SEM image with the region marked as “III” in the Figure 4 obtained for the sintered Mg_63_Zn_30_Ca_4_Au_3_ alloy. The image features the transcrystalline fracture.

**Figure 6:**
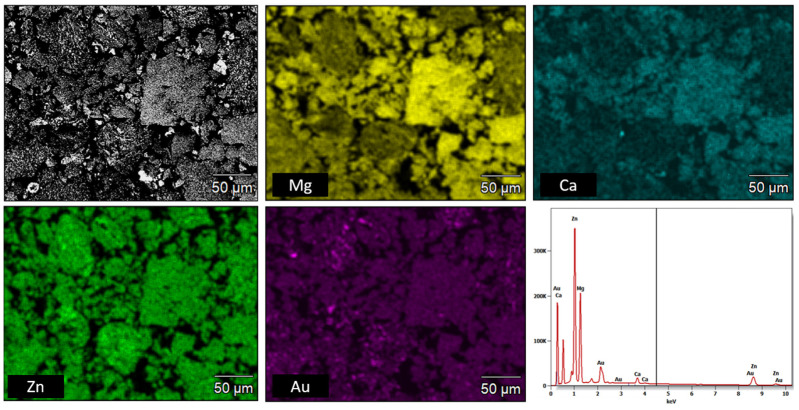
EDS chemical composition map for the powder sample of the Mg_63_Zn_30_Ca_4_Au_3_ alloy.

**Figure 7:**
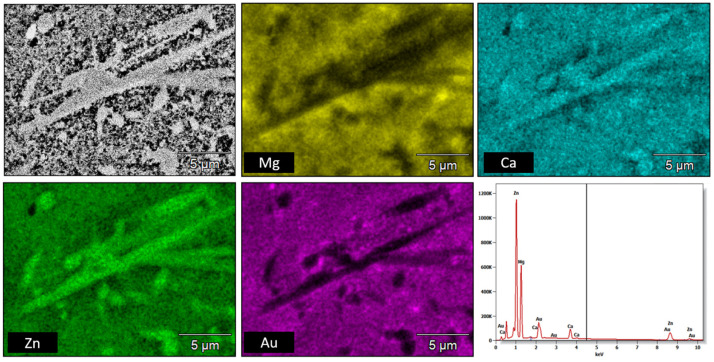
EDS chemical composition map for the sintered sample of the Mg_63_Zn_30_Ca_4_Au_3_ alloy.

**Figure 8:**
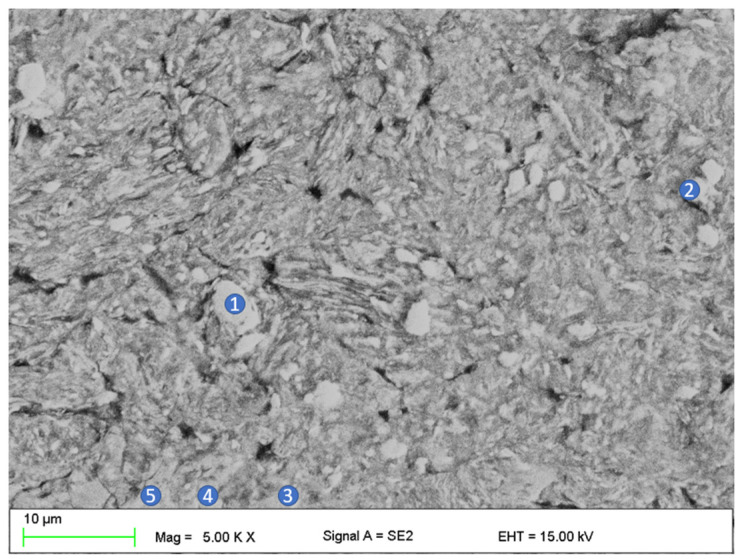
The SEM image featuring MgZn_2_ phase regions in the powder sample. The numbers in the image refer to the EDS analysis results which are showcased in Table 4.

**Figure 9:**
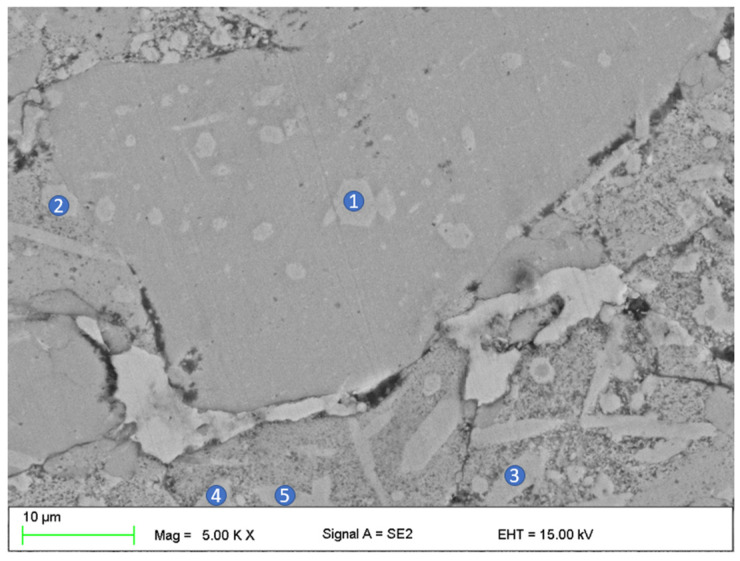
The SEM image featuring MgZn_2_ phase regions in the sintered sample. The numbers in the image refer to the EDS analysis results which are showcased in Table 5.

SEM micrographs presented in Figure 4 were taken from sample pieces after compression tests to feature their fracture morphology, as well as the sintered samples chemical composition obtained via EDS. In Figure 4, the SEM image of the position marked with a black arrow as I, II, and III at higher magnification, as well as refined areas selected for the EDS test (Table 3), are presented. In the SEM image (Figure 4 and Figure 5), the cracks and brittle nature of the sinter was visible. The morphology of the sample indicates that the sinter had a brittle fracture of intercrystalline structure (Figure 4) as well as transcrystalline character (Figure 5) after compressive test. The results for the numbered areas are featured in Table 3. The values indicate a homogeneous distribution of the alloying elements, as they do not differ from the assumed chemical composition of Mg_63_Zn_30_Ca_4_Au_3_ alloy. Moreover, the homogeneity of the distribution is further supported by the distribution maps featured in Figure 6 and Figure 7 for both powder and sintered samples. Thus, it may be concluded that the homogeneity obtained via MA is retained after the SPS process. Furthermore, the MgZn_2_ phase marked and seen in Figure 8 (points 3 ÷ 5) and 9 (points 1 ÷ 5) is retained during the sintering process, which is further supported by the EDS (see Figure 8 and Figure 9, Table 4 and Table 5); the obtained results clearly indicate a higher content of Zn over Mg. These claims are reflected in the XRD patterns in Figure 1 and Figure 2, where the MgZn2 phase was identified. The presence of this phase is beneficial for the alloy; according to the literature, it is known that it improves the stability and corrosion resistance of the material compared to pure Mg or Zn [30,31].

### 3.3. Particle Size Distribution

**Figure 10:**
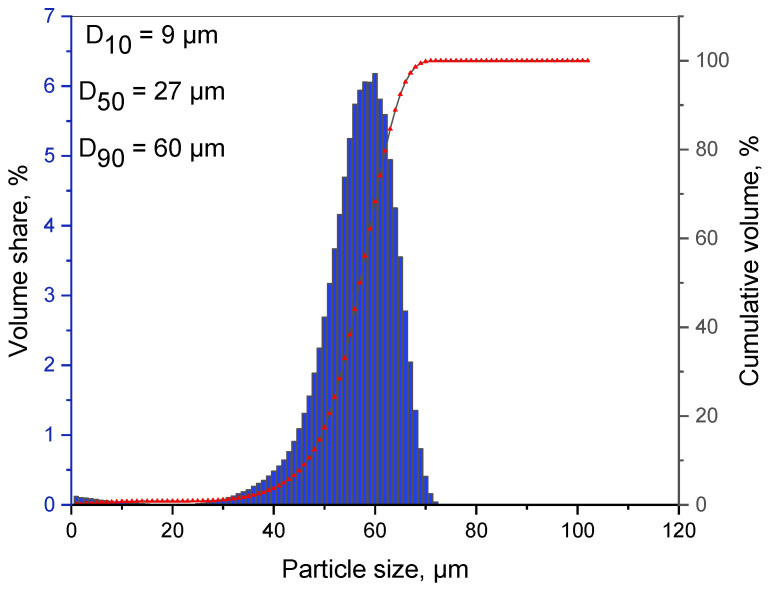
Granulometry chart of the Mg–Zn–Ca–Au powder alloy after 13 h of milling.

Figure 10 represents the data obtained from the granulometry test and the average particle size. The results of the laser particle size measurement of the sample after 13 h of milling (Figure 10) showed that the average particle size was 27 μm. Produced grains are in the range from 0 to about 75 μm (10% grains smaller than 9 μm ± 1 μm, 50% grains smaller than 27 μm ± 3 μm, and 90% grains smaller than 60 μm ± 10 μm). The granular distribution of the powder after 13 h of milling has the maximum of about 65 μm (unimodal distribution). The relatively narrow distribution curve can imply that the process of MA has reached the set condition, demonstrating the state of balance between the mechanisms of joining and fragmentation (Figure 10). This particle size distribution can be advantageous to being further processed.

### 3.4. Microhardness

**Figure 11:**
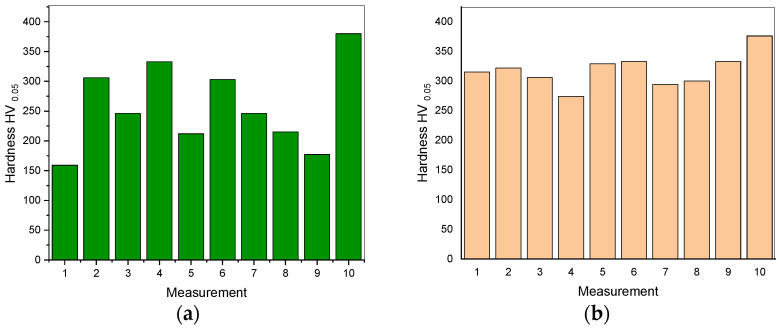
The microhardness results for (**a**) Mg–Zn–Ca–Au powder alloy after 13 h of milling and (**b**) Mg–Zn–Ca–Au sintered alloy.

The results of both powder and sintered samples are shown in the Figure 11a,b, respectively. The average values were 258 ± 71 HV for the powdered sample and 318 ± 28 HV for the sintered one. The hardness of the sintered sample is much higher than that of the powders.

The hardness saturation is connected to the measurement of the mean average values, which comprise the hardness of different phases present in the prepared alloys—that is why there are clear differences between both powdered and sintered samples. In the MA process, non-equilibrium phases are created. On the other hand, during SPS, thermal processes are activated, leading to the formation of equilibrium phases. To assess the hardness of constituent phases properly, nano-indentation testing would be necessary, which is a consideration for the future research.

### 3.5. Corrosion Resistance

**Figure 12:**
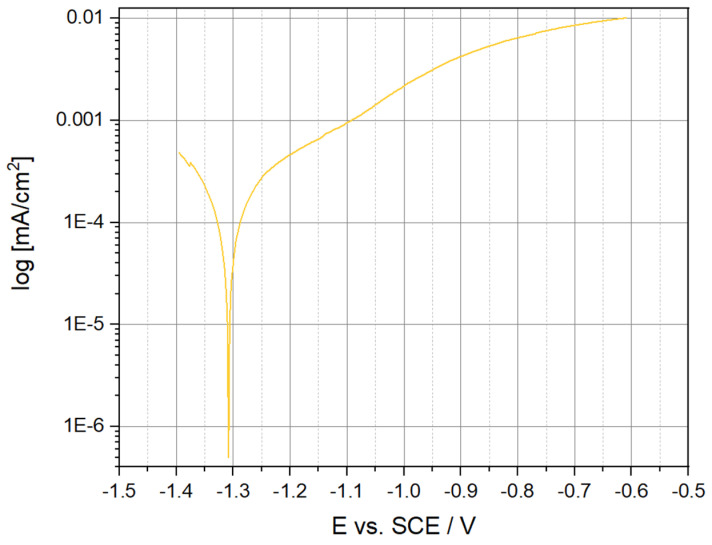
Polarization curve for the Mg–Zn–Ca–Au sintered alloy.

The mean value of the corrosion potential of E_corr_ for the sintered Mg_63_Zn_30_Ca_4_Au_3_ alloy sample after 13 h of milling is −1.353 V. The determined mean value of the corrosion current density i_corr_ and the polarization resistance R_p_ (Stern’s method) for the sintered alloy sample after 13 h MA are i_corr_ = 338 μA/cm^2^ and R_p_ = 77 Ω∙cm^2^. Polarization curves recorded for the sintered sample are shown in Figure 12.

**Figure 13:**
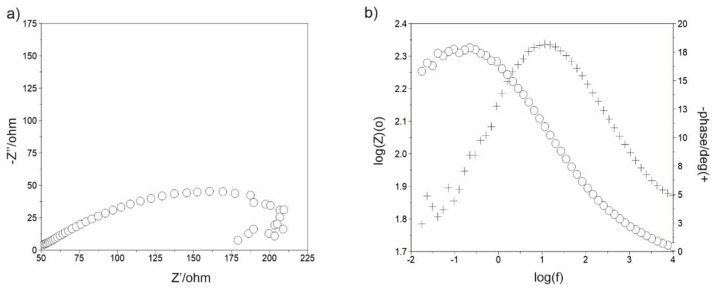
Impedance spectra obtained for Mg–Zn–Ca–Au sintered alloy. (**a**) Nyquist diagram, (**b**) Bode’s diagram. The symbols in (**b**) refer to the amplitude (circles) and phase spectra (“plus” sign).

**Figure 14:**
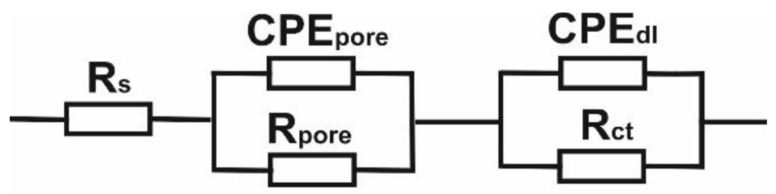
Electrical equivalent circuit of the corrosion system for samples made of Mg–Zn–Ca–Au sintered alloy.

To obtain information on the electrochemical properties of the sintered Mg–Zn–Ca–Au alloy surface, tests were carried out with the use of electrochemical impedance spectroscopy (EIS), see Figure 13. For the analysis of impedance spectra of corrosion systems of Mg alloy–Ringer’s solution, substitute electrical systems were used. On this basis, the parameters of the elements of the electrical equivalent circuit describing the corrosion system were determined. This method allowed for the analysis and interpretation of the processes and phenomena occurring at the interface: material–Ringer’s solution. The impedance spectra obtained for the sintered Mg alloy samples were interpreted by comparison to the equivalent electrical system, which indicates the presence of two sub-layers: dense inner and porous outer (two time constants shown in the graph), where R_s_ is the solution resistance (Ringer’s solution), R_pore_—the solution resistance in the pore, CPE_pore_—capacitance of the porous (top) layer, and R_ct_ and CPE_dl_—the double-layer resistance and the double-layer capacitance, respectively (Table 6). The use of two constant-phase elements in the electrical equivalent circuit had a positive effect on the quality of matching the experimentally determined curves. Substitute electrical systems were used to analyze the impedance spectra of corrosion systems of Mg alloy–Ringer’s solution. On this basis, the parameters of the elements of the equivalent electrical circuit describing the corrosion system were determined and are shown in Figure 14. This method allowed for the analysis and interpretation of the processes and phenomena occurring at the interface: material–Ringer’s solution [32,33]. The determined mean value of the corrosion current density—i_corr_, the polarization resistance—R_p_, and the mean value of the corrosion potential—E_corr_ for the Mg_63_Zn_30_Ca_4_Au_3_ sintered alloy sample after 13 h of MA are 338 μA/cm^2^, 77 Ω∙cm^2^, and −1.353 V, respectively (Figure 12). When comparing these values to the AZ31 and AZ91 commercially available alloys, although the corrosion potential of Mg_63_Zn_30_Ca_4_Au_3_ is lower, the corrosion rates resulting from the Rp and i_corr_ values indicate worse corrosion resistance in similar environments [34,35,36]. AZ31 and AZ91 alloys immersed in Ringer’s solution have E_corr_ values of −1.498 and −1.527 V, respectively, as compared to the −1.353 V of the Mg_63_Zn_30_Ca_4_Au_3_ alloy [35]. The shift of E_corr_ to the more negative value means the metal is more thermodynamically susceptible to corrosion. However, the corrosion current i_corr_ of the Au_3_ alloy is much greater than that of AZ31 and AZ91 commercial alloys. The i_corr_ value is proportional to the corrosion rate. This issue is reflected as well in R_p_ values, where AZ31 and AZ91 values are one order of magnitude greater [35,36].

The values of the compressive strength amounted to 216 MPa and is lower than in the Mg–Zn–Ca alloy (264–300 MPa) [22]. The Young’s modulus amounted to 2530 MPa. For comparison, the sintered Mg_65_Zn_30_Ca_4_Gd_1_ alloy has a compressive strength and a Young’s modulus of 308 MPa and 4443 MPa, respectively [37]. In turn, the AZ31 alloy used in the automotive industry is characterized by a Young’s modulus of 4500 GPa [38]. However, the compressive strength (216 MPa) of the sintered alloy is comparable to the bone properties.

## 4. Conclusions

- The phase composition of the powders obtained by mechanical synthesis after 13 h of grinding consists of MgZn_2_ and Mg_3_Au intermetallic phases; a Mg-based solid solution with a residue of unreacted Zn. The phase analysis of the alloys after sintering indicates the retention of these phases and the formation of an additional Mg_7_Zn_3_ phase. The formation of the MgZn_2_ phase, confirmed by both XRD and EDS analysis, is advantageous due to the improvement in stability and overall corrosion resistance of the alloy.
- The chemical composition is homogeneous, with individual regions showing a higher concentration of magnesium in the places where intermetallic phases separate. Despite the structural and phase changes during the sintering process, the chemical distribution remains the same or very similar. Moreover, the phases resultant from the milling do not change their character in the sintering process. Additionally, the Mg_7_Zn_3_ phase appears, which features the desired chemical and mechanical properties.
- The microhardness of the sintered samples (HV0.05 = 318 ± 28) is higher than that of the powder particles (HV0.05 = 258 ± 71).
- The average compressive strength of the alloy is 216 MPa, the Young’s modulus is 2530 MPa, and the fracture morphology after compression is characteristic of brittle crystalline materials.
- The corrosion resistance tests indicate the ability to degrade the Mg–Zn–Ca–Au alloy in Ringer’s solution. The recorded potentiodynamic curves are characterized by the absence of a passive area. The value of the charge transfer resistance determined in the impedance tests also confirms the fact that the double layer formed as a result of contact with the solution is not an effective protective barrier against the effects of Ringer’s solution.
- Biodegradability is closely related to the corrosion resistance. The determined mean value of the corrosion current density—i_corr_, the polarization resistance—R_p_, and the mean value of the corrosion potential—E_corr_ for the Mg_63_Zn_30_Ca_4_Au_3_ sintered alloy sample after 13 h of MA are 338 μA/cm^2^, 77 Ω∙cm^2^, and 1.353 V, respectively. Mg_63_Zn_30_Ca_4_Au_3_ alloy, when compared to the commercially available alloys (i.e., AZ31, AZ91), has more stable corrosion potential, although its general corrosion resistance is weaker. The analyzed alloy constitutes a prospective biodegradable material, although it requires further research.

## Figures and Tables

**Table 1:** Structural parameters, crystallite sizes and lattice strain for the main phases present in the Mg–Zn–Ca–Au sintered alloy.

Mg–Zn–Ca–Au 13 h	Mg(X), X = Zn, Ca, Au	MgZn_2_	Mg_3_Au
Theoretical	(ICDD PDF4 + Card: 00-035-0821) a = 3.2094 [Å] c = 5.2112 [Å] Space group: P6_3_/mmc Crystallographic System: Hexagonal	(ICDD PDF4 + Card: 04-008-7744) a = 5.2210 [Å] c = 8.5670 [Å] Space group: P6_3_/mmc Crystallographic System: Hexagonal	(ICDD PDF4 + Card: 04-003-5362) a = 4.6600 [Å] c = 8.4880 [Å] Space group: P6_3/_mmc Crystallographic System: Hexagonal
Refined (RR) a/c [Å]	a = 3.2263(6) c = 5.2472(9)	a = 5.6389(9) c = 8.6214(5)	a= 4.7255(1) c= 8.5278(6)
Crystallite Size D [Å]	360	350	103
Lattice Strain η [%]	0.69	0.62	0.32

**Table 2:** EDS results from the area marked in Figure 3.

Wt. %	Mg	Si	Ca	Zn	Au
1	34.8	0.2	5.3	47.6	12.0
2	26.3	-	3.8	57.6	12.3
**At. %**	**Mg**	**Si**	**Ca**	**Zn**	**Au**
1	60.6	0.3	5.6	47.6	2.6
2	51.0	-	4.5	41.6	3.0

**Table 3:** EDS results from the areas marked in Figure 4.

Wt. %	Mg	Ca	Zn	Au
1	40.6	3.5	43.6	12.2
2	40.5	4.4	41.7	13.5
3	42.9	1.6	41.5	14.0
4	42.8	3.8	40.6	12.8
5	32.4	1.4	61.6	4.6
6	44.6	3.1	40.6	11.8
**At. %**	**Mg**	**Ca**	**Zn**	**Au**
1	67.2	3.6	26.8	2.5
2	67.1	4.4	25.7	2.8
3	57.1	1.5	40.4	1.0
4	69.3	3.7	24.4	2.6
5	70.3	1.6	25.3	2.8
6	70.8	2.9	24.0	2.3

**Table 4:** EDS results from the areas marked in Figure 8.

Wt. %	Mg	Ca	Zn	Au
1	0.7	0.1	47.7	51.5
2	5.2	0.4	38.6	55.8
3	14.9	2.1	72.7	10.2
4	19.1	1.4	74.1	5.4
5	18.2	0.4	78.2	3.2
**At. %**	**Mg**	**Ca**	**Zn**	**Au**
1	2.9	0.3	71.3	25.5
2	19.6	0.8	53.8	25.8
3	33.5	2.9	60.7	2.8
4	39.7	1.7	57.2	1.4
5	38.0	0.5	60.6	0.8

**Table 5:** EDS results from the areas marked in Figure 9.

Wt. %	Mg	Ca	Zn	Au
1	17.1	6.9	76.1	-
2	18.2	7.0	74.8	-
3	19.7	-	80.3	-
4	18.8	-	81.2	-
5	16.4	6.8	76.8	-
**At. %**	**Mg**	**Ca**	**Zn**	**Au**
1	34.5	8.4	57.1	-
2	36.2	8.5	55.3	-
3	39.8	-	60.2	-
4	38.4	-	61.6	-
5	33.4	8.4	58.2	-

**Table 6:** Corrosion parameter values: E_OCP_, R_s_, R_pore_, CPE_pore_, R_ct_, CPE_dl_ for sintered Mg–Zn–Ca–Au alloy.

Corrosion Parameters							
E_OCP_, V	R_s_, Ω/cm^2^	R_pore_, Ω/cm^2^	CPE_pore_		R_ct_, Ω/cm^2^	CPE_dl_	
			Y_0_, Ω^−1^cm^−2 s−n^	n		Y_0_, Ω^−1^cm^−2 s−n^	n
−1.299	48	79	0.1295 × 10^−3^	0.80	83	0.8433 × 10^−3^	0.55

## Data Availability

Data available on request.
